# Does the Intersectionality of Race/Ethnicity and Type 2 Diabetes Increase the Odds of a Cervical Cancer Diagnosis? A Nested Case–Control Study of a Florida Statewide Multisite EHR Database

**DOI:** 10.3390/healthcare11131863

**Published:** 2023-06-27

**Authors:** Rahma S. Mkuu, Jaclyn M. Hall, Zhanna Galochkina, Hee Deok Cho, Stephanie A. S. Staras, Ji-Hyun Lee, Yi Guo, Choeeta Chakrabarti, Sable Bowman Barrow, Selena Ortega, Daniel M. Avery, John Higginbotham, Jala Lockhart, Elizabeth A. Shenkman

**Affiliations:** 1 Department of Health Science, The University of Alabama, Tuscaloosa, AL 35487, USA; 2 Department of Health Outcomes and Biomedical Informatics, The University of Florida, 2199 Mowry Road, Gainesville, FL 32611, USAhdcho5016@ufl.edu (H.D.C.); sablebowman@ufl.edu (S.B.B.);; 3 Division of Quantitative Sciences, University of Florida Health Cancer Center, The University of Florida, 2033 Mowry Road, Gainesville, FL 32610, USA; z.galochkina@ufl.edu; 4 Department of Biostatistics, The University of Florida, 2004 Mowry Road, Gainesville, FL 32603, USA; 5 Department of Anthropology, Florida State University, 2035 E Paul Dirac Drive Suite 206, Tallahassee, FL 32310, USA; 6 College of Community Health Sciences, The University of Alabama, 211 Peter Bryce Boulevard, Tuscaloosa, AL 35401, USA

**Keywords:** type 2 diabetes, cervical cancer, social vulnerability, intersectionality, race/ethnicity

## Abstract

Cervical cancer and Type 2 Diabetes (T2D) share common demographic risk factors. Despite this, scarce research has examined the relationship between race/ethnicity, having T2D, and cervical cancer incidence. We analyzed statewide electronic health records data between 2012 and 2019 from the OneFlorida+ Data Trust. We created a 1:4 nested case–control dataset. Each case (patient with cervical cancer) was matched with four controls (patients without cervical cancer) without replacement by year of encounter, diagnosis, and age. We used conditional logistic regression to estimate the unadjusted and adjusted odds ratios (ORs) and 95% confidence intervals (CIs) to examine the association between race/ethnicity, T2D, and cervical cancer incidence. A total of 100,739 cases and 402,956 matched controls were identified. After adjusting for sociodemographic characteristics, non-Hispanic Black women with T2D had higher odds of cervical cancer compared with non-Hispanic White women with T2D (OR: 1.58, 95% CI 1.41–1.77). Living in a rural area, having Medicaid/Medicare insurance, and having high social vulnerability were associated with higher odds of having a cervical cancer diagnosis. Our findings imply the need to address the higher burden of cervical cancer diagnosis among non-Hispanic Black women with T2D and in underserved populations.

## 1. Introduction

In the United States (U.S.), diabetes has been diagnosed in approximately 11.3% of the population, with Type 2 Diabetes (T2D) accounting for 90–95% of all diagnosed cases [1]. Several cancers among women, including colorectal, breast, and endometrial, have been associated with T2D [2,3]. The association between T2D and cancers that primarily affect women is hypothesized to occur because of the interaction between hormones, inflammation, and insulin-like growth factors associated with both conditions [4,5]. The excess insulin and insulin-like growth factor associated with T2D is suspected to drive the increased risk of developing cancer and having poor cancer prognosis among individuals with T2D [4,5]. Cervical cancer and T2D also share similar demographic risk factors. Demographic characteristics of populations who are more likely to experience deleterious outcomes from both conditions include being from a historically underrepresented group (e.g., Black or Hispanic), having low social-economic status (SES), and living in rural areas [6,7,8]. For example, nationally, compared with non-Hispanic White women, Black women have higher rates of new cervical cancer diagnosis, are more likely to be diagnosed with an advanced stage of cervical cancer, and are more likely to die from cervical cancer [9,10,11]. Data from the 2011–2016 National Health and Nutrition Examination Surveys show that compared with non-Hispanic White people (12.1%, 95% CI 11.0–13.4%), non-Hispanic Black individuals (22.1%, 95% CI 19.6–24.7%) have a significantly higher weighted prevalence of diabetes (adjusted for age and sex) [12]. Despite similar associated factors between T2D and cervical cancer, scarce research has examined the relationship between having T2D, race/ethnicity, and receiving a cervical cancer diagnosis [4].

The national trends of cervical cancer and diabetes are mirrored in the state of Florida. The state of Florida has the third largest population and is home to the second largest Black population in the country [13]. In Florida, the prevalence of T2D among Black women is 14% compared with 10% among White women [14]. Additionally, compared with non-Hispanic White women, non-Hispanic Black women in Florida have higher rates (per 100,000) of new cervical cancer diagnosis (11.2 vs. 8.6), advanced-stage cervical cancer diagnosis (6.4 vs. 4.6), and cervical cancer mortality (3.8 vs. 2.5) [15].

Cervical cancer is preventable through Human Papillomavirus vaccination or concordance with screening guidelines, which encourages early detection of abnormalities before cells become cancerous [16]. However, medically underserved women, women living in poverty, and women living in rural areas are more likely to miss cervical cancer screening [8,17]. Several studies have shown that women with T2D have lower cervical cancer screening rates [18,19,20], and have higher mortality rates from cervical cancer compared with women without T2D [21,22]. Given the similar sociodemographic trends between cervical cancer and T2D, there is a need to examine the relationship between T2D and receiving a cervical cancer diagnosis while controlling for sociodemographic factors (race/ethnicity and SES). A nuanced understanding of how T2D, race/ethnicity, and cervical cancer diagnosis are associated has the potential to influence cancer prevention approaches for populations with a higher burden of T2D.

The primary aim of this paper is to elucidate the relationship between T2D, race/ethnicity, and cervical cancer diagnosis and examine whether the intersectionality of race/ethnicity and T2D plays a role in cervical cancer diagnosis in a statewide clinical sample. The secondary aim of this paper is to examine the relationship between sociodemographic factors (rurality; insurance; social vulnerability) with cervical cancer diagnosis.

## 2. Materials and Methods

### 2.1. Data Source

We analyzed electronic health records (EHR) data from the OneFlorida+ Data Trust, a repository containing real-world data for 19.21 million patients in Florida that reflects the demographic characteristics of the state by age, race, and ethnicity [23]. The data trust is a secure and centralized repository where 11 health care system partners throughout the state of Florida submit data using the Patient Centered Outcomes Research Institute’s Common Data Model [24]. A data use agreement between the University of Florida and the Florida Medicaid program allows the data trust to contain linked Medicaid enrollment and claims data [23].

### 2.2. Participants

We derived the study population of women in Florida aged 25–65 years from the OneFlorida+ patient data between 2012 and 2019.

### 2.3. Case–Control Selection and Inclusion Criteria

Because we were interested in examining whether exposure to Type 2 Diabetes increases the risk of a later outcome of cervical cancer, a nested case–control design was more appropriate. The benefits of the nested case–control design included cost and effort efficiency, ability to examine biological precursors of the disease compared with the full cohort study design, and avoiding the implicit meaning of ‘causality’. We performed a matching algorithm to create a 1:4 nested case–control dataset. Cases were defined as the patients with a registration of cervical cancer in the period 1 January 2012 until 31 December 2019. For each case, the pool of controls consisted of all eligible individuals without a diagnosis of cervical cancer before 31 December 2019. We matched cases and controls on year of encounter/diagnosis and age. An optimal matching algorithm was used to match individuals without replacement, accounting for the longitudinal nature of the registry data [25]. An additional advantage of using optimal matching and nested case–control design was reduced selection bias given that the cases and controls were selected from the same population. The original data had 3,063,626 eligible individuals. The total sample after applying the nested case–control algorithm was 503,695, with 100,739 of the sample being cervical cancer cases.

### 2.4. Variables

We identified patients with a cervical cancer and T2D diagnosis by using International Classification of Diseases (ICD)-9, Ninth Revision, Clinical Modification or ICD-10-CM diagnosis codes [26]. We excluded patients who had a cervical cancer diagnosis date that preceded the first encounter date with a T2D diagnosis code in the study period.

The outcome variable was being diagnosed with cervical cancer during the period of 1 January 2012–31 December 2019. Explanatory variables included prior T2D status (at any time before 31 December 2019), race/ethnicity, rurality, insurance status, and the social vulnerability index (SVI). The following combinations of race and ethnicity were used: (1) Non-Hispanic White (NHW); (2) Non-Hispanic Black (NHB); (3) Non-Hispanic Other/Asian/Unknown (NHOAU); (4) Hispanic (All Races); (5) Unknown Ethnicity (All Races).

We used the Rural Urban Continuum Codes (RUCC) to classify geographic locations by degree of rurality and labeled geographic locations as metropolitan (when RUCC code = 1, 2, 3) and non-metropolitan (rural) (when RUCC code = 4, 5, 6, 7, 8, 9). The RUCC code for a county of residence was taken from each patient’s listed address in the year 2018 [27]. We assumed non-relocation of patients.

For insurance, in case multiple payer codes were available per any patient’s encounter, we selected the top one in order of priority (the rule is described below), then selected insurance information from a record of the earliest encounter during the relevant matched year (see matching algorithm description), and based on the first digit of that payer code, we assigned the insurance group type. Prioritization rule of insurance type used: first, “Insured/non-government”, second “Medicare”, third “Medicaid/other government”, fourth “Uninsured”, and the last “Unknown”. For example, if a patient had non-government and Medicare, we assigned the patient to have non-government insurance.

The Centers for Disease Control and Prevention (CDC) uses 15 social factors, including poverty, lack of vehicle access, and crowded housing to rank census tracts by a value of social vulnerability. Each individual’s 9-digit zip code information was used to categorize social vulnerability, which was reported on a scale of 0 to 1, with higher scores indicating greater vulnerability [28]. We divided the SVI scores into 4 quartiles, with Q3 and Q4 representing highest vulnerability.

### 2.5. Statistical Analysis

The demographic characteristics of the patients in the nested case–control study were summarized and compared using descriptive statistics such as frequency and proportion for discrete variables and mean and standard deviation or median and range for continuous variables. We used conditional logistic regression to estimate the unadjusted and then adjusted odds ratios (ORs) and associated 95% confidence intervals (CIs) to investigate the association between cervical cancer and T2D. The pre-specified variables of interest as potential covariates included race/ethnicity, rurality, insurance type, and social vulnerability. No imputations for missing data were considered and we treated missing data as the unknown category. All tests were two-sided, and the alpha level was 0.05. All statistical analyses were conducted using R version 4.2.1.

### 2.6. Data Availability

The data for this study were made available to the authors after undergoing scientific review through the OneFlorida+ Coordinating Center. Researchers submitted for ethics review and obtained IRB approval (IRB #IRB202101001) before receiving the HIPAA-limited data sets, which restrict types of identifiable protected health information (e.g., birthdates, dates of service, zip codes).

## 3. Results

### 3.1. Characteristics of the Study Patients

Our total sample comprised 503,695 patients, with 402,956 having no cervical cancer and 100,739 having a positive cervical cancer diagnosis. In the overall sample, more than half, 58%, of patients were between the ages of 25 and 39 years, 33.2% were non-Hispanic White, 17% were non-Hispanic Black, and 25.8% were Hispanic. In the total patient sample, 96.3% of patients resided in metropolitan areas and 24.7% and 19.8% of patients were at the two highest levels of social vulnerability (SVI Q4 and SVI Q3, respectively). Medicaid and other government insurance covered 49.9% of patients, while 31.3% were insured by private insurance. Demographic characteristics of the case–control dataset including a description of demographic characteristics by cervical cancer presence are provided in Table 1.

### 3.2. Prevalence of T2D

Between 2012 and 2019, the proportion of T2D among the OneFlorida+ case–control dataset of 503,695 women in Florida aged 25–65 years was 2.4%. Among the sample without a cervical cancer diagnosis (n= 402,956), the proportion of T2D was 2.3%. The proportion of T2D among women with a cervical cancer diagnosis (N = 100,739) was 2.8%.

### 3.3. Association between Cervical Cancer and T2D

Univariable conditional logistic regression results revealed that women with T2D had 24% higher odds of having cervical cancer, compared with women without T2D (OR: 1.24, 95% CI 1.19–1.29). Non-Hispanic Black women had 55% higher odds of having cervical cancer compared with non-Hispanic White women (OR: 1.55, 95% CI 1.52–1.58). Increased SVI was associated with higher odds of having a cervical cancer diagnosis. The odds of having cervical cancer among women with the highest vulnerability (SVI quartile = 4) was 59% higher compared with those with the lowest vulnerability (SVI quartile = 1) (OR: 1.59, 95% CI 1.55–1.63). The univariable logistic regression model revealed the variables associated with significantly higher odds of cervical cancer diagnosis included T2D, race/ethnicity, rurality, insurance, and SVI (Table 2).

Further, a multivariable conditional logistic regression analysis included the pre-specified variables: diabetes status, race/ethnicity, rurality, insurance, and SVI. The model showed that intersections of the variables race/ethnicity and diabetes, rurality, public insurance, and high social vulnerability were significant predictors of cervical cancer. After adjusting T2D status by other sociodemographic variables, the effect of T2D on cervical cancer diagnosis vanished (OR: 1.00, 95% CI 0.95–1.05). Holding all other predictor variables constant, Non-Hispanic Black women had 34% higher odds of having cervical cancer compared with non-Hispanic White women (OR: 1.34, 95% CI (1.31–1.36). Compared with private/non-governmentally insured women, women insured by Medicaid had 117% significantly higher odds of being diagnosed with cervical cancer (OR: 2.17, 95% CI 2.12–2.21); the same findings were observed for women insured by Medicare, who had 115% higher odds of being diagnosed with cervical cancer compared with privately insured women (OR: 2.15, 95% CI 2.06–2.24). The odds of having cervical cancer increased with an increasing level of SVI. Women with the highest vulnerability (SVI = 4) had 25% higher odds of having cervical cancer compared with women with the lowest vulnerability (SV1 = 1) (OR: 1.25, 95% CI 1.22–1.29). Women from rural counties had 39% significantly higher odds of cervical cancer compared with women from metropolitan counties (OR: 1.39, 95% CI 1.34–1.44). Findings from multivariable logistic regression analysis are provided in Table 3.

**Figure 1:**
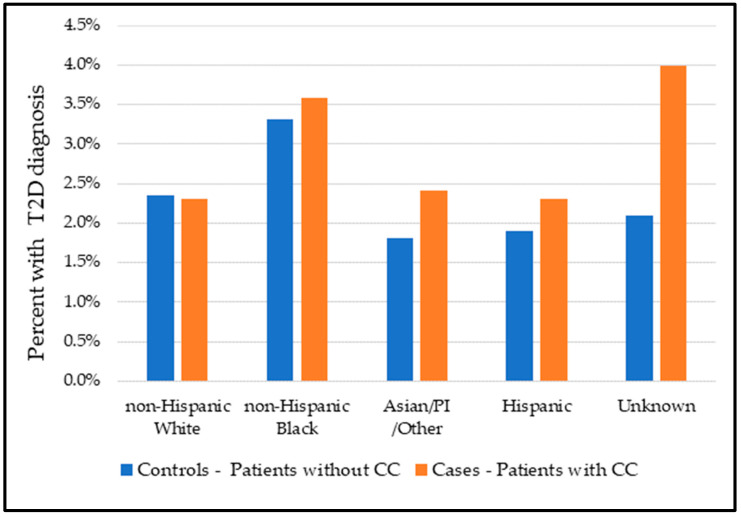
Proportion of Type 2 Diabetes (T2D): comparison between controls (patients without cervical cancer (CC)) and cases (patients with CC).

We ran a multivariable model comparing the intersection of race/ethnicity and T2D diagnosis controlling for rurality, insurance status, and SVI. Figure 1 illustrates the rationale of running the interaction model as the percent of individuals diagnosed with diabetes and cervical cancer in both cases and the control was not quite similar, especially for non-Hispanic Black females.

The multivariable model comparing the intersection of race/ethnicity and T2D diagnosis controlling for rurality, insurance status, and SVI showed that all variables were significant predictors of cervical cancer holding other predictor variables constant. Non-Hispanic Black women with T2D had 58% higher odds of cervical cancer compared with non-Hispanic White women with T2D (OR: 1.58, 95% CI 1.41–1.77). Non-Hispanic White women without T2D had 21% higher odds of cervical cancer compared with non-Hispanic White women with T2D (OR: 1.21, 95% CI 1.12–1.31). In the model, women living in rural areas had 39% higher odds of cervical cancer compared with women in metropolitan areas (OR: 1.39, 95% CI 1.34–1.44). Women with Medicaid or Medicare insurance had higher odds of receiving a cervical cancer diagnosis, compared with women with private insurance. As the SVI level increased, the odds of cervical cancer increased. Women with the highest social vulnerability (SVI = 4) had 25% higher odds of cervical cancer compared with women at the lowest level of social vulnerability (SVI = 1) (OR: 1.25, 95% CI 1.22–1.29). Findings from the multivariable logistic regression analysis examining the intersection of race/ethnicity and T2D on cervical cancer diagnosis are provided in Table 4.

## 4. Discussion

To our knowledge, our study is the first to examine the relationship between T2D, race/ethnicity, and cervical cancer diagnosis using EHR data. Overall, we observed that T2D was associated with 24% higher odds of cervical cancer in the univariate model; however, after adjusting for sociodemographic factors, T2D was not a significant predictor of cervical cancer diagnosis. Underlying differences in sociodemographic characteristics, particularly having Black race, Medicaid or Medicare insurance, low socioeconomic status, and rurality, appear to be more important predictors of disparities in cervical cancer diagnosis and not diabetes independently.

Given that we found no direct association between T2D and cervical cancer, our study adds to the limited studies in the U.S. and conflicting findings in the literature. Unlike this study’s use of T2D diagnosis codes, a Mendelian randomized study using genetic T2D predisposition data and data from the Breast Cancer Association Consortium and UK Biobank found higher odds of cervical cancer with genetic predisposition to T2D (OR: 1.08, 95% CI 1.01–1.15) [29]. Our study did not examine the relationship between length of T2D diagnosis and cervical cancer diagnosis given that the scope of our focus was to delineate the relationship between having diabetes and receiving a cervical cancer diagnosis. Nevertheless, a Canadian retrospective study comparing the cervical cancer risk of patients with and without diabetes found that women newly diagnosed with T2D (within 3 months) had a higher risk of receiving a cervical cancer diagnosis. However, the relationship was not significant 3 months to 10 years after the T2D diagnosis index date [30]. Furthermore, a recent umbrella review of observational and Mendelian randomized studies reported a weak association between T2D and associated biomarkers with cervical cancer [31]. To our knowledge, our study is the first to examine the relationship between T2D and cervical cancer using a robust clinical database of patients in the U.S. More research is needed given the inconsistent and scarce studies highlighting the association between T2D and cervical cancer.

The main and key clinically relevant finding in our study was that after adjusting for sociodemographic characteristics, non-Hispanic Black women with T2D had 58% higher odds of cervical cancer diagnosis compared with non-Hispanic White women with T2D. In the U.S., non-Hispanic Black women have higher rates of cervical cancer diagnosis compared with other groups. National surveillance data from 2019 released by the U.S. Cancer Statistics Working Group in June 2022 shows that the rate of new cervical cancers per 100,000 women among Black women is 8.2 compared with 7.3 among White women [11]. Diabetes may play a role in elevating the prevalence of cervical cancer diagnosis among Black women. Additionally, T2D is an additional factor increasing the odds of cervical cancer risk in Black women because women with T2D have lower rates of cervical cancer screening compared with women without T2D [19,20]. Cervical cancer screening is associated with early detection of precancerous cervical changes, resulting in the prevention of cervical cancer [16]. Black women with T2D may not be receiving cervical cancer screening due to persistent barriers including competing priorities and responsibilities, financial barriers, experiences of discrimination in clinical settings, fear of diagnosis, social stigma, history of trauma, mistrust of the health care system, underrepresentation of Black providers, and other barriers [32,33]. Another explanation for our findings is screening prioritization. Previous findings have also reported that women with diabetes and other chronic conditions are not receiving recommended cervical cancer screenings and hypothesize that uncontrolled chronic conditions are prioritized over cervical cancer screenings [34,35,36]. Providers report that prioritization of existing uncontrolled conditions during clinical visits over preventative care as one barrier to providing cervical cancer guideline concordant screening to patients with existing chronic conditions [37]. Non-Hispanic Black people with T2D are more likely to experience uncontrolled levels of T2D compared with non-Hispanic White people [38]. Therefore, non-Hispanic Black women with T2D may prioritize T2D control over preventative screenings during doctor’s visits, leading to their higher odds of being diagnosed with cervical cancer. Further research should be undertaken to investigate cervical cancer diagnosis among non-Hispanic Black women with T2D.

Interestingly, we found that non-Hispanic White women without T2D had a 21% higher odds of cervical cancer diagnosis compared with non-Hispanic White women with T2D. More frequent interaction with the health care system to manage T2D among White women with T2D may explain our findings. White women with T2D may have more opportunities for early detection of abnormalities before they develop cancer because they may see their health care providers more frequently. However, a recent study using nationally representative data from the Behavioral Risk Factor Surveillance System (BRFFS) and TeenVaxView found that between 2001 and 2019, White women had a greater annual increase in missing cervical cancer screenings and had lower HPV vaccination rates compared with other racial groups [10]. Medical mistrust and vaccine hesitancy explain lower rates of cervical cancer screening and HPV vaccination among White women [39,40]. Non-Hispanic White women without T2D in our study may have less contact with the health care system than those with T2D, resulting in lower screening rates and, therefore, increasing their odds of cervical cancer diagnosis.

Similar to previous studies, our findings show that factors which limit access to health care including living in rural areas and living in a community of higher social vulnerability were significantly associated with higher odds of cervical cancer [20,21]. In a study conducted by Spencer and colleagues [41] projecting cervical cancer incidence in the U.S. through the year 2070, results show that low-poverty counties will reach the goal of near elimination of cervical cancer 14 years earlier than high poverty counties (2029 vs. 2043). Addressing persistent disparities in access to health care that are driven by area poverty is key to addressing preventable cervical cancers among socially vulnerable women.

The higher odds of cervical cancer among women with Medicaid/Medicare insurance in our study may be explained by the association between cervical cancer and lack of access to early screening. Women who have never been screened or have not been screened in the last 5 years make up half of all new cervical cancer diagnoses in the United States [42]. In Florida, uninsured women can be screened for cervical cancer and receive treatment through the Florida Breast and Cervical Cancer Early Detection Program (FBCCEDP) that is administered by the CDC [43]. The FBCCEDP collaborates with Medicaid to provide treatment and referral to women who are diagnosed with cervical cancer [44]. The higher rate of cervical cancer among women with Medicaid may be driven by use of FBCCEDP, and by previously uninsured women being newly insured through Medicaid during pregnancy. The Medicaid and Medicare programs are important safety programs for underserved women and future research should consider the programs as an avenue to improve cervical cancer outcomes of populations served by the programs.

This study has some limitations. Our study population was derived from a clinical database, and therefore excludes women who have not accessed care at one of the OneFlorida+ clinical partners. Therefore, our findings cannot be generalized to women with no access to care, or to woman being served by clinical facilities not partnering with OneFlorida+. Although the data used in this study are robust with respect to the number of health systems that contribute their data, our findings may be underestimated because of under-reporting or women seeking care at institutions that do not report data to OneFlorida+. We did not have access to patient T2D diagnosis dates that were diagnosed before 2012, as OneFlorida+ data collection commenced in 2012. The proportion of women with T2D in this study is significantly lower than reported proportions in other studies in Florida. This limitation of our observational study is likely due to not having diagnosis information for patients who may have been diagnosed before 2012. Therefore, no causal inferences can be made and results should be interpreted with caution. We used the first mention of T2D in EHR records to identify whether women were diagnosed with diabetes before receiving a cervical cancer diagnosis. We did not control for other chronic conditions in the model. For this study, we used histology-based classification (CIN1, CIN2,3) to define cervical cancer diagnosis and did not analyze by cervical cancer stage. A recent study suggests using HPV genotype and other biomarkers that are more parsimonious in detecting cervical cancer in research [45]. We plan to examine whether data partners in OneFlorida+ use the recommended classifications in future research. Notwithstanding the limitations, our study has several strengths. Our study adds to the limited examinations of the relationship between T2D and cervical cancer. We used a robust statewide dataset that has been used in previous studies to characterize the landscape of several health conditions, including obesity and hypertension, in the state of Florida [46,47].

## 5. Conclusions

In conclusion, our study found that when intersected with race/ethnicity, T2D is a significant predictor of getting a cervical cancer diagnosis. Non-Hispanic Black women who have T2D had a significantly higher odds of cervical cancer diagnosis compared with non-Hispanic White women with T2D. Black women with T2D and social vulnerability may be especially vulnerable to cervical cancer. Sociodemographic characteristic of living in a rural area, having high social vulnerability, and having Medicaid/Medicare insurance were associated with higher odds of having a cervical cancer diagnosis, stressing the need to continue addressing cervical cancer disparities in underserved populations. The findings highlight the importance of addressing poverty-driven health care access challenges to increase cervical cancer prevention in underserved populations. Moreover, our findings also have important implications for the clinical community, as they highlight the need to improve cervical cancer prevention among all women, particularly underserved Black women with T2D.

## Figures and Tables

**Table 1:** Descriptive statistics.

Characteristic	Overall, N = 503,695	CC ^1^-Negative, N = 402,956	CC-Positive, N = 100,739
**Age (years)**			
<30	113,025 (22.4%)	90,420 (22.4%)	22,605 (22.4%)
30–39	179,485 (35.6%)	143,588 (35.6%)	35,897 (35.6%)
40–49	103,855 (20.6%)	83,084 (20.6%)	20,771 (20.6%)
50+	107,330 (21.3%)	85,864 (21.3%)	21,466 (21.3%)
**Type 2 Diabetes Status**			
No Type 2 Diabetes	491,591 (97.6%)	393,685 (97.7%)	97,906 (97.2%)
Type 2 Diabetes Positive	12,104 (2.4%)	9271 (2.3%)	2833 (2.8%)
**Race/Ethnicity**			
Non-Hispanic White	167,165 (33.2%)	130,129 (32.3%)	37,036 (36.8%)
Non-Hispanic Black	85,483 (17.0%)	59,523 (14.8%)	25,960 (25.8%)
Non-Hispanic Other/Asian/Unknown	18,443 (3.7%)	16,035 (4.0%)	2408 (2.4%)
Hispanic (All Races)	130,099 (25.8%)	105,330 (26.1%)	24,769 (24.6%)
Unknown Ethnicity (All Races)	102,505 (20.4%)	91,939 (22.8%)	10,566 (10.5%)
**Type 2 Diabetes by Race/Ethnicity**			
Non-Hispanic White _T2Dpos ^2^	3919 (0.8%)	3067 (0.8%)	852 (0.8%)
Non-Hispanic Black _T2Dpos	2906 (0.6%)	1976 (0.5%)	930 (0.9%)
Non-Hispanic Other/Asian/Unknown _T2Dpos	349 (0.1%)	291 (0.1%)	58 (0.1%)
Hispanic _T2Dpos	2581 (0.5%)	2009 (0.5%)	572 (0.6%)
Unknown Ethnicity _T2Dpos	2349 (0.5%)	1928 (0.5%)	421 (0.4%)
Non-Hispanic White _T2Dneg ^3^	163,246 (32.4%)	127,062 (31.5%)	36,184 (35.9%)
Non-Hispanic Black _T2Dneg	82,577 (16.4%)	57,547 (14.3%)	25,030 (24.8%)
Non-Hispanic Other/Asian/Unknown _T2Dneg	18,094 (3.6%)	15,744 (3.9%)	2350 (2.3%)
Hispanic_T2Dneg	127,518 (25.3%)	103,321 (25.6%)	24,197 (24.0%)
Unknown Ethnicity _T2Dneg	100,156 (19.9%)	90,011 (22.3%)	10,145 (10.1%)
**Social Vulnerability**			
SVI Q ^4^ 1(Lowest Vulnerability)	50,136 (10.0%)	39,810 (9.9%)	10,326 (10.3%)
SVI Q2	79,388 (15.8%)	61,925 (15.4%)	17,463 (17.3%)
SVI Q3	99,791 (19.8%)	75,620 (18.8%)	24,171 (24.0%)
SVI Q4 (Highest Vulnerability)	124,480 (24.7%)	88,430 (21.9%)	36,050 (35.8%)
SVI Unknown	149,900 (29.8%)	137,171 (34.0%)	12,729 (12.6%)
**Rurality**			
Metropolitan	485,298 (96.3%)	389,495 (96.7%)	95,803 (95.1%)
Non-Metropolitan (Rural)	18,397 (3.7%)	13,461 (3.3%)	4936 (4.9%)
**Insurance**			
Private Insurance	157,595 (31.3%)	139,623 (34.6%)	17,972 (17.8%)
Medicare	16,103 (3.2%)	11,385 (2.8%)	4718 (4.7%)
Medicaid/Other Government Insurance	251,304 (49.9%)	181,735 (45.1%)	69,569 (69.1%)
Uninsured	19,634 (3.9%)	18,940 (4.7%)	694 (0.7%)
Unknown	59,059 (11.7%)	51,273 (12.7%)	7786 (7.7%)

^1^ CC—cervical cancer. ^2^ T2DPos—Type 2 Diabetes positive. ^3^ T2Dneg—no Type 2 Diabetes. ^4^ SVI Q—social vulnerability index quartile.

**Table 2:** Univariable conditional logistic regression results showing odds of cervical cancer diagnosis by sociodemographic characteristic and Type 2 Diabetes.

Variable	Term	N	OR	LCL	UCL	*p*. Value	Global. *p*
T2D by Race/Ethnicity	Reference: Non-Hispanic White–T2DPos ^1^						<0.001
	Non-Hispanic Black_T2Dpos	2906	1.70	1.53	1.90	<0.001	
	Non-Hispanic Other/Asian_T2Dpos	349	0.72	0.54	0.97	0.0303	
	Hispanic_T2Dpos	2581	1.03	0.91	1.16	0.6428	
	Unknown Ethnicity_T2Dpos	2349	0.78	0.68	0.89	<0.001	
	Non-Hispanic White_T2Dneg ^2^	163,246	1.07	0.99	1.15	0.1061	
	Non-Hispanic Black_T2Dneg	82,577	1.64	1.52	1.78	<0.001	
	Non-Hispanic Other/Asian _T2Dneg	18,094	0.56	0.51	0.61	<0.001	
	Hispanic_T2Dneg	127,518	0.88	0.82	0.95	0.0018	
	Unknown Ethnicity _T2Dneg	100,156	0.42	0.38	0.45	<0.001	
Type 2 Diabetes	Reference: No T2D						<0.001
	Type 2 Diabetes Positive	12,104	1.24	1.19	1.29	<0.001	
Race/Ethnicity	Reference: Non-Hispanic White	167,165					<0.001
	Non-Hispanic Black	85,483	1.55	1.52	1.58	<0.001	
	Non-Hispanic Other/Asian/Unknown	18,443	0.53	0.50	0.55	<0.001	
	Hispanic (All Races)	130,099	0.83	0.82	0.85	<0.001	
	Unknown Ethnicity (All Races)	102,505	0.40	0.39	0.41	<0.001	
Rurality	Reference: Metropolitan						<0.001
	Non-Metropolitan (Rural)	18,397	1.49	1.44	1.54	<0.001	
Insurance	Reference: Private Insurance						<0.001
	Medicare	16,103	3.11	2.99	3.24	<0.001	
	Medicaid/Other Government	251,304	3.18	3.12	3.24	<0.001	
	Uninsured	19,634	0.29	0.27	0.31	<0.001	
	Unknown	59,059	1.18	1.15	1.22	<0.001	
Social Vulnerability	Reference: SV1 ^3^ Q1						<0.001
	SVI Q2	79,388	1.10	1.07	1.13	<0.001	
	SVI Q3	99,791	1.25	1.21	1.28	<0.001	
	SVI Q4	124,480	1.59	1.55	1.63	<0.001	
	SVI Unknown	149,900	0.35	0.34	0.36	<0.001	

^1^ T2DPos—Type 2 Diabetes Positive. ^2^ T2Dneg—No Type 2 Diabetes. ^3^ SVI—social vulnerability index quartile.

**Table 3:** Multivariable conditional logistic regression showing odds of cervical cancer diagnosis after adjusting for sociodemographic characteristics and T2D.

Variable	Term	N	OR	LCL	UCL	*p.* Value	Global. *p*
Type 2 Diabetes	Reference: T2D Negative						
	T2D Positive	12,104	1.00	0.95	1.05	0.9588	0.9588
Race/Ethnicity	Reference: Non-Hispanic White						
	Non-Hispanic Black	85,483	1.34	1.31	1.36	<0.001	<0.001
	Non-Hispanic Other/Asian/Unknown	18,443	0.90	0.86	0.94	<0.001	
	Hispanic (All Races)	130,099	0.66	0.65	0.68	<0.001	
	Unknown Ethnicity (All Races)	102,505	0.54	0.53	0.56	<0.001	
Rurality	Reference: Metropolitan						
	Rural	18,397	1.39	1.34	1.44	<0.001	<0.001
Insurance	Reference: Private Insurance						
	Medicare	16,103	2.15	2.06	2.24	<0.001	<0.001
	Medicaid/Other Gov	251,304	2.17	2.12	2.21	<0.001	
	Uninsured	19,634	0.27	0.25	0.29	<0.001	
	Unknown	59,059	0.90	0.88	0.93	<0.001	
Social Vulnerability	Reference: SVI ^1^ = 1						
	SVI Q2	79,388	1.02	0.99	1.05	0.1536	<0.001
	SVI Q3	99,791	1.06	1.04	1.09	<0.001	
	SVI Q4	124,480	1.25	1.22	1.29	<0.001	
	SVI Unknown	149,900	0.50	0.48	0.51	<0.001	

^1^ SVI—social vulnerability index quartile.

**Table 4:** Multivariable logistic regression results showing odds of cervical cancer diagnosis by Type 2 Diabetes.

Variable	Term	N	OR	LCL	UCL	*p.* Value	Global. *p*
T2D by Race and Ethnicity	Reference: Non-Hispanic White and T2DPos ^1^)	3919	-	-	-		<0.001
	Non-Hispanic Black and T2Dpos	2906	1.58	1.41	1.77	<0.001	
	Non-Hispanic Other/Asian/Unknown and T2Dpos	349	1.19	0.88	1.61	0.2712	
	Hispanic and T2Dpos	2581	0.88	0.77	1.00	0.0419	
	Unknown Ethnicity and T2Dpos	2349	0.89	0.78	1.02	0.0945	
	Non-Hispanic White and T2Dneg ^2^	163,246	1.21	1.12	1.31	<0.001	
	Non-Hispanic Black and T2Dneg	82,577	1.61	1.49	1.75	<0.001	
	Non-Hispanic Other/Asian/Unknown and T2Dneg	18,094	1.08	0.98	1.18	0.1095	
	Hispanic and T2Dneg	127,518	0.80	0.74	0.87	<0.001	
	Unknown Ethnicity and T2Dneg	100,156	0.65	0.60	0.70	<0.001	
Rurality	Reference: Metropolitan	485,298	-	-	-		<0.001
	Non-Metropolitan (Rural)	18,397	1.39	1.34	1.44	<0.001	
Insurance	Reference: Private Insurance	157,595	-	-	-		<0.001
	Medicare	16,103	2.15	2.07	2.24	<0.001	
	Medicaid/Other Government	251,304	2.16	2.12	2.21	<0.001	
	Uninsured	19,634	0.27	0.25	0.29	<0.001	
	Unknown	59,059	0.90	0.88	0.93	<0.001	
Social Vulnerability	Reference: SVI ^3^ Q1	50,136	-	-	-		<0.001
	SVI Q2	79,388	1.02	0.99	1.05	0.1481	
	SVI Q3	99,791	1.07	1.04	1.10	<0.001	
	SVI Q4	124,480	1.25	1.22	1.29	<0.001	
	SVI Unknown	149,900	0.50	0.48	0.51	<0.001	

^1^ T2DPos—Type 2 Diabetes positive. ^2^ T2Dneg—no Type 2 Diabetes. ^3^ SVI—social vulnerability index quartile.

## Data Availability

The datasets generated during and analyzed in this current study are available from the OneFlorida+ Dataset upon request.
